# Genome Sequence of *Lactobacillus plantarum* EGD-AQ4, Isolated from Fermented Product of Northeast India

**DOI:** 10.1128/genomeA.01122-13

**Published:** 2014-01-09

**Authors:** Asifa Qureshi, Yogeshwari Itankar, Ramkrishna Ojha, Manabendra Mandal, Anshuman Khardenavis, Atya Kapley, Hemant J. Purohit

**Affiliations:** Environmental Genomics Division, National Environmental Engineering Research Institute (CSIR-NEERI), Nagpur, Indiaa; Tezpur University, Assam, Indiab

## Abstract

We present a draft genome sequence of *Lactobacillus plantarum* strain EGD-AQ4, isolated from nonalcoholic fermented bamboo shoot products of Northeast India. The size of the draft genome sequence is the largest among all the reported genome sequences of *Lactobacillus plantarum*, thus enabling the exploration of new gene clusters involved in various functional and probiotic attributes.

## GENOME ANNOUNCEMENT

*Lactobacillus plantarum* is a highly versatile lactic acid bacterium found in various ecological niches, such as fermented vegetable, meat, and dairy products and the gastrointestinal tract. It has been reported that fermented bamboo shoot food products of Northeast India provide various health benefits and probiotic effects (1). *Lactobacillus plantarum* strain EGD-AQ4 was isolated from such nonalcoholic fermented bamboo shoot food products. This bacterial isolate was found to possess some probiotic features, like bile tolerance, acid tolerance, and resistance to various antibiotics, such as vancomycin, tetracycline, rifampin, penicillin, streptomycin, gentamycin, nalidixic acid, and polymyxin B.

*Lactobacillus plantarum* EGD-AQ4 was grown in Man, Rogosa, and Sharpes (MRS) broth at 35°C under anaerobic conditions. The bacterial culture grown overnight was harvested and total DNA was prepared using a Fast DNA spin kit (MP Biomedicals, France). DNA purity was measured by the A_260_-to-A_280_ ratio with the NanoDrop 1000 spectrophotometer (Thermo Scientific). The genome was sequenced using the Illumina MiSeq platform.

A total of 447,074 reads were generated, and by use of the GC Assembler/CLC Genomics Workbench, 442,671 reads were assembled into 52 scaffolds with maximum scaffold size of 570,301 and a minimum size of 548. The total genome (sequence) length is 3,420,019.The genome was annotated using the NCBI Prokaryotic Genomes Automatic Annotation Pipeline (PGAAP) and was independently analyzed on the Rapid Annotations using Subsystems Technology (RAST) server (2).

The information contained in the draft genome comprised 3,420,019 bp of sequence data, with a mean GC content of 46.4%, and the data cover a total of 3,135 genes, 37 pseudogenes, 60 tRNAs, 3 rRNAs, and 3,035 proteins. The sequence of the 16S rRNA gene (1,504 bp) of *Lactobacillus plantarum* strain EGD*-*AQ4 is 100% identical to those of *L. plantarum* strain IMAU10168 (NCBI accession no. GU125610.1) and *L. plantarum* strain ZJ316 (NCBI accession no. CP004082.1) (3).

While exploring the preliminary annotation data of the *Lactobacillus plantarum* strain EGD-AQ4 genome for genes related to bile tolerance, we found two copies of the choloylglycine hydrolase (EC 3.5.1.24) gene in contig 17 (bp 30040 to bp 29054), and in contig 8 (bp 129191 to bp 130207). These genes are responsible for hydrolyzing conjugated bile salts. which enhances bacterial tolerance to bile salts (3, 5). The genome also showed the presence of 22 genes along with the key enzyme pyruvate formate lyase (PFL), responsible for mixed acid fermentations. Genes encoding beta-lactamase and various other proteins involved in multidrug resistance were found in contigs 16 and 11 and contigs 1 and 8, respectively. Genes involved in various oxidative stress responses were also present (4). Genbank has reported only one draft genome of *Lactobacillus plantarum* WCFS1, the size of which is 3.35 Mb (5). Knowledge of the whole-genome sequence of *L. plantarum* strain EGD-AQ4 will help to increase our understanding of several unknown gene clusters that may be involved in other probiotic attributes.

### Nucleotide sequence accession number.

This whole-genome shotgun project of *L. plantarum* has been deposited at DDBJ/EMBL/GenBank under the accession no. AVAQ00000000.
